# Cutaneous Leishmaniasis Vaccination: A Matter of Quality

**DOI:** 10.3389/fimmu.2016.00151

**Published:** 2016-04-21

**Authors:** Paula Mello De Luca, Amanda Beatriz Barreto Macedo

**Affiliations:** ^1^Laboratório de Imunoparasitologia, Instituto Oswaldo Cruz, FIOCRUZ, Rio de Janeiro, Brazil; ^2^Department of Pathology, Division of Microbiology and Immunology, University of Utah School of Medicine, Salt Lake City, UT, USA

**Keywords:** vaccines, multifunctionality, T cell response, *Leishmania amazonensis*, *Leishmania braziliensis*

## Abstract

There have been exhaustive efforts to develop an efficient vaccine against leishmaniasis. Factors like host and parasite genetic characteristics, virulence, epidemiological scenarios, and, mainly, diverse immune responses triggered by *Leishmania* species make the achievement of this aim a complex task. It is already clear that the induction of a Th1, pro-inflammatory response, is important in the protection against *Leishmania* infection. However, many questions must still be answered to fully understand *Leishmania* immunopathology, especially regarding *Leishmania*-specific Th1 response induction, regulation, and persistence. A large number of *Leishmania* antigens able to induce pro-inflammatory response have been selected so far, but none of them demonstrated efficiency in protection assays. A possible explanation is that CD4 T cells display marked heterogeneity at a single-cell level especially regarding the production of Th1-defining cytokines and multifunctionality. It has been established in the literature that Th1 cells undergo a differentiation process, which can generate cells with diverse phenotypes and survival capabilities. Despite that, only a few studies evaluate this heterogenic response and the amount of multifunctional CD4 T cells induced by *Leishmania* vaccine candidates, missing what can be a crucial point in defining a correlate of protection after vaccination. Moreover, most of the knowledge involving the development of cutaneous leishmaniasis (CL) vaccines comes from the mouse model of infection with *Leishmania major*, which cannot be fully applied to New World Leishmaniasis. For this reason, the immune response triggered by infection with New World *Leishmania* species, as well as vaccine candidates, need further studies. In this review, we will reinforce the importance of evaluating the quality of immune response against *Leishmania*, using a multiparametric analysis in order to understand better this complex host-parasite interaction, discussing the differences in the responses triggered by different New World *Leishmania* species, as well as the impact on the development of an effective vaccine against CL.

## Introduction

World Health Organization (WHO) has classified Leishmaniasis among the tropical neglected, emerging, and uncontrolled diseases that affect mainly poor regions around the Globe. The disease is endemic in 88 countries (72 are developing countries) with approximately 350 million individuals at risk of contracting the disease and an annual incidence of 1.5–2 million new cases (1). Its prevention has been based on control of vectors and animal reservoirs in countries where the disease has a zoonotic transmission, combined with chemotherapy of infected individuals where the disease possesses anthroponotic features. However, control of reservoir hosts and vectors is difficult due to operational issues, making the development of an effective and affordable vaccine against Leishmaniasis a highly desirable task.

The history of *Leishmania* vaccination dated from twentieth century, in which live, virulent parasites were inoculated in healthy individuals in a process called “Leishmanization.” The practice was banned because of safety concerns due to development of non-healing lesions and immunosuppression (2). First generation vaccines using whole-killed *Leishmania* promastigotes replaced Leishmanization and were tested as vaccines against cutaneous leishmaniasis (CL) and visceral leishmaniasis (VL) (3, 4). Second and third generation vaccines were also developed, based on the defined synthetic or recombinant subunits and DNA, respectively. Despite many years of efforts in identifying a great number of antigens (5) and advances in vaccine technologies, there does not yet appear to be a vaccine candidate capable of delivering the level of protection needed for disease control.

The localized form of CL, specially the one caused by the Old World specie *Leishmania major*, is a self-healing disease, usually characterized by a state of at least partial immunity against reinfection, demonstrating that prevention through prophylactic vaccination is feasible. On the other hand, although recovery from infection with the New World specie *Leishmania braziliensis* gives firm resistance to homologous challenge, *Leishmania amazonensis* infection does not provide protection against a subsequent challenge with *L. braziliensis*, or other *Leishmania* species from the subgenus *Viannia* (6, 7). Until now, there has been no consistent data, particularly in humans, indicating that recovery from a primary infection with *L. amazonensis* gives complete resistance to a homologous challenge.

The fact that there is not yet an efficient vaccine against Leishmaniasis, especially one that could protect against different species simultaneously, leads us to consider that a better understanding of immune response in *Leishmania* pathogenesis is still needed, taking into consideration the various species that cause different clinical manifestations of the disease. Among the reasons that can be pointed out to explain our failure in developing a vaccine against CL, particularly against American cutaneous leishmaniasis, is the fact that we are still far from fully understand the mechanisms of healing and of memory responses generated after *Leishmania* infections as well as how to evaluate this responses. Far from giving the answers, this review focuses on the current advances in T cell memory knowledge and the differences observed between the immune responses induced after infection with different *Leishmania* species, particularly between *L. braziliensis* and *L. amazonensis*.

## American Tegumentary Leishmaniasis: Beyond the Th1/Th2 Paradigm

American tegumentary leishmaniasis (ATL) is endemic in Latin America and the most common species involved are: *L. braziliensis*, *Leishmania guyanensis*, *Leishmania panamensis* (all from the genus *Viannia*), *L. amazonensis*, and *Leishmania mexicana* (both from the subgenus *Leishmania*). Unlike Old World CL, usually characterized by subclinical or self-healing cutaneous lesions, the infection by ATL causing species can lead to uncontrolled parasite replication, producing non-healing cutaneous, mucosal, or even visceral disease (1) (Table 1).

**Table 1:** **Major human American cutaneous leishmaniasis causing species and their clinical manifestations**.

Species	Subgenus	Clinical forms	Leishmanin skin test	Key cytokines involved in pathology
*L. braziliensis*	*Viannia*	LCL	++	IFN-γ+++, IL-10++
		Disseminated CL	+++	IFN-γ+, IL-10++
		ML	++++	IFN-γ++++, IL-10+
*L. guyanensis*	*Viannia*	LCL	++	IFN-γ++, IL-10++
*L. panamensis*	*Viannia*	LCL	++	IFN-γ++, IL-10++
		ML	++++	IFN-γ++++, IL-10+
*L. amazonensis*	*Leishmania*	LCL	±	IFN-γ+, IL-10+
		Borderline CL	−	IFN-γ++, IL-10++
		DCL	−	IFN-γ±, IL-10++++
*L. mexicana*	*Leishmania*	LCL	±	IFN-γ++, IL-10++
		DCL	−	IFN-γ±, IL-10++++

*LCL, localized cutaneous leishmaniasis; ML, mucosal leishmaniasis; DCL, diffuse cutaneous leishmaniaisis*.

*−, Absence*.

*+, Presence; +, low; ++, moderate; +++, high; ++++, very high*.

Human infection with *L. braziliensis* leads to a broad spectrum of clinical, immunological, and histopathological manifestations, varying from self-healing cutaneous lesions to the severe and destructive clinical form named mucocutaneous leishmaniasis (ML) (8–10). The localized cutaneous form (LCL) usually manifests as one or a few ulcers with elevated borders and sharp craters that increase rapidly in size and heal slowly without treatment (11). *L. braziliensis* can also cause disseminated leishmaniasis, in which up to hundreds of lesions erupt as a result of hematogenous spread of parasite (12, 13). *L. amazonensis* has also been isolated from patients with diverse clinical forms, such as simple CL lesions to diffuse cutaneous leishmaniasis (DCL) (14) and was also implicated in borderline disseminated CL, an intermediate form of disease (15). Patients with DCL are often resistant to chemotherapy, have negative leishmanin skin test (LST), and low or negative responses after *Leishmania* antigen-specific stimulation *in vitro* but remain responsive for other unrelated antigens, such as tuberculin (8).

For many years, murine CL models have been used to elucidate the cell types, cytokines, signal transduction cascades, and mechanisms needed for parasite control and clinical resolution of the disease. Since *Leishmania* is an obligate intracellular parasite, the protective immunity is associated with a cell-mediated immune response. Indeed, studies in the murine model have been helping to elucidate the immunological pathways that are responsible for resistance or susceptibility to *Leishmania* and were responsible for the description of the CD4 T cells Th1/Th2 dichotomy. It is well accepted that protective immunity against *Leishmania* parasites is mediated by a type 1, pro-inflammatory response, and most of the early studies, particularly on *L. major* infection, largely defined the Th1/Th2 paradigm of resistance/susceptibility to infection and the role of interleukin 12 (IL-12) and IL-4, respectively, in driving Th1 and Th2 cell development (16, 17). On the other hand, in human *L braziliensis* infection, some evidences suggest that higher percentage of activated IFNγ+ producing T CD4+ lymphocytes are associated with larger lesions (18), and an exacerbated Th1 response is observed in ML (19). The polarized CD4 lymphocyte response detected in the murine *L. major* model is not so evident in the human Leishmanasis, and the importance of IL-4 as a primary mediator of susceptibility to *Leishmania* infection is not corroborated by clinical trials (19). Indeed, in DCL (the most severe form of human ATL), the main cytokine associated with immunosuppression and pathology it is not IL-4 but IL-10 (20–22).

The vast majority of experimental CL studies come from the murine model of infection with *L. major*, although the disease outcome in inbred strains of mice differs among *Leishmania* species. While C57BL/6 and C3H mice are resistant to infection with *L. major*, they develop chronic lesions when infected with *L. amazonensis* while BALB/c mice are highly susceptible to *L. major* and *L. amazonensis* infection, but develop self-limited lesions when infected with *L. braziliensis* (16). Little information has been generated in the murine model regarding ATL causing *Leishmania* species. Although some data have been published with *L. braziliensis* and *L. amazonensis* infection, the protocols are heterogeneous with respect to the stage of parasite used (stationary phase or metacyclic promastigotes) and the inoculation route (subcutaneous or intradermal), making it difficult to compare the results obtained (23–26).

Even though we still lack a reliable, largely accepted, and utilized murine model for ATL, much progress was made in understanding the mechanisms involved in human pathology. However, many questions are still unanswered, especially those related to the immunological mechanisms leading to lesions healing and natural resistance to infection and cross-protection, as well as to the induction, regulation, and persistence of *Leishmania*-specific T cell response.

## CD4 Immune Response and Memory

The goal of vaccination is the development of immunological memory, classically defined as the ability of the immune system to respond more effectively and faster to a pathogen previously encountered. In the late twentieth century, memory T cells were divided into central memory (TCM) and effector memory cell (TEM) populations, based on the expression of different cell surface markers (27). TCM cells constitutively express CCR7 and CD62L and are found in T cell areas of secondary lymphoid organs where they are able to proliferate and differentiate into effector cells in response to antigenic stimulation. TEM cells downregulate the expression of CCR7, have heterogeneous expression of CD62L, and are able to migrate to inflamed tissues, and have immediate effector functions (27, 28). One study in murine *L. major* infection demonstrated the importance of two populations of memory CD4 T cells in the protection against reinfection. While effector CD4+ T cells are lost in the absence of parasites, the central memory CD4+ T cells are kept and become tissue-homing effector T cells to mediate protection, suggesting that central memory T cells should be the targets for vaccines against *Leishmania* (29). The same group recently identified the presence of skin tissue *Leishmania*-specific resident memory T cells, and indicated the necessity of these cells, together with circulating memory T cells, for the success of a vaccine (30).

The induction of memory T cells was also evaluated in patients with CL. In patients healed form *L. major* infection both TEM IFN-γ producing cells (CD4+CD45RO+CD45RA−CCR7−) and *Leishmania*-reactive IL-2 producing TCM cells (CD4+CD45RO+CD45RA−CCR7+) were observed after “*in vitro*” stimulation with *Leishmania* soluble antigen (SLA), suggesting that both populations might play a role in protective recall immune response against reinfection (31). On the other hand, the majority of *L. braziliensis*-healed CL and ML patients did not produce IFN-γ “*in vitro*” after SLA stimulation, but are still responsive “*in vivo*” to LST. A positive LST was found in 87.5% of CL and 100% of ML cured individuals who did not produce IFN-γ, and in the individuals that maintains SLA-specific IFN-γ production, the main source of the cytokine was effector memory CD4+ T cell (32).

Usually, *L. braziliensis* patients healed from CL lesions should be monitored for approximately 5 years to rule out of the possibility of relapses or the development of metastatic mucosal lesions (33, 34). In one study where healed *L. braziliensis* CL patients were grouped according to the time elapsed since the end of therapy, a regulated leishmanial-specific response appeared to emerge only about 2 years after initial contact with the parasite. *Ex vivo* analyses showed a contraction for both CD4 and CD8 TEM compartments in patients with long-time elapsed after clinical cure (2–5 years). However, after “*in vitro*” SLA stimulation, they exhibit a recall response with expansion of TEM cells (35).

CD4 T cells also present different capacities to develop into memory cells based on their cytokine production (36), and the quality of a Th1 immune response has been related with a differentiation spectrum based on the production of three cytokines: IFN-γ, IL-2, and TNF-α (37). Cells that enter this differentiation processes are, at first, single producers of IL-2 or double producers of IL-2/TNF-α, but are negative for IFN-γ. They can be classified as central memory cells, since are long-lasting cells able to respond quickly to a second antigen encounter. Te other pole of this spectrum is IFN-γ single-positives cells that are short-lived, terminal effector cells (36, 37). From one pole to the other, a variety of phenotypes can be found, including multifunctional CD4 T that are triple positives for IFN-γ, IL-2, and TNF-α (37). Interestingly, the amount of IFN-γ produced by multifunctional cells is much higher than the amount produced by double- or single-positive cells (38, 39). The IL-2 produced by those cells together with the high production of IFN-γ and TNF-α give to multifunctional CD4 T cells the remarkable capacity to possess optimal effector functions and proliferation.

CD4+ T cells not always go through each possible stage of differentiation and after antigen recognition, an IL-2 single-positive cell can go straight to the IFN-γ single-positive effector phenotype, particularly if the stimulus is strong (36). Thus, it is possible that a vaccine candidate can elicit an immune response predominantly composed by effector cells, and fail to induce long, last protection against infection. A promising vaccine candidate should be able to induce multifunctional T cells that are able to proliferate and generate memory and effector cells.

In past years, the majority of studies designed to evaluate possible immunogens against *Leishmania* infection utilized the production of IFN-γ by antigen-specific T cells as the main factor to predict protection. However, it is clear that the quality and the magnitude of a T-cell response measured by a single parameter do not reflect its full functional potential which may be the reason why vaccines that reached phase III trials failed to protect against *Leishmania* infection (40–42). In 2007, the first compelling evidence for the importance of multifunctional Th1 cells in mediating protection against Leishmaniasis revealed, after immunization with various vaccine formulations encoding specific *L. major* antigens, a strong correlation between the generation of multifunctional CD4 T cells and the degree of protection observed after a subsequent challenge (38). Intriguingly, the best degree of protection and the higher percentage of multifunctional T cells were observed in animals that healed primary lesions and were reinfected (“live vaccination”). Afterward, this approach started to be utilized by many other research groups to characterize immune correlates of protection after infection or after immunization against CL and VL (Table 2) (39, 43–51), but only two concerned ATL causing species (39, 51). In all of them, protection was demonstrated to be associated with the induction of multifunctional T cells among other double producers or with TNF-α producing cells (either TNF-α single-positives or TNF-α/IL-2 and TNF-α/IFN-γ double positive cells) (45–47, 51).

**Table 2:** **Multifunctional T cells analysis on *Leishmania* infection and vaccination**.

Specie	Model	CD4 T cell phenotype associated with cure or protection	Reference
*L. major*	C57BL/6	IFN+TNF+IL-2+	Darrah et al. (38)
*L. major*	C57BL/6 and human	IFN+TNF+IL-2+	Darrah et al. (50)
*L. major*	Balb/c	IFN+TNF+L-2+, IFN+NF+, and TNF+	Sánchez-Sampedro et al. (45)
*L. major*	Balb/c	IFN+TNF+ and IFN+IL-2+	Hugentobler et al. (49)
*L. amazonensis* and *L. braziliensis*	Human	IFN+TNF+IL-2+	Macedo et al. (39)
*L. donovani*	Balb/c	IFN+TNF+L-2+, IFN+TNF+, and IFN+IL-2+	Dey et al. (48)
*L. donovani*	Balb/c	TNF+IL-2+ and IFN+TNF+	Guha et al. (46)
*L. donovani*	Balb/c	IFN+TNF+IL-2+, IFN+TNF+, and IFN+IL-2+	Guha et al. (47)
*L. amazonensis*	Balb/c	TNF+IL-2+ and TNF+	Nico et al. (51)
*L. major*	Balb/c and C57BL/6	IFN+TNF+IL-2+	Matos et al. (43)
*L. major*	Human	IFN+TNF+IL-2+	Lakhal-Naouar et al. (44)

## *L. Amazonensis* Versus *L. Braziliensis*: Differences on Quality of Immune Response

It has already been reported that patients infected with parasites from the subgenus *Viannia* (as *L. braziliensis*) display higher T cell responses (evaluated by proliferation and IFN-γ production) to *Leishmania* crude antigens than *L. amazonnsis*-infected patients, and that *L. amazonensis-*infected patients also have stronger responses to *L. braziliensis* than to *L. amazonensis* antigens *in vitro*, before and after therapy (52).

Vaccine candidates formulated with *L. braziliensis* total extract have been tested against Canine VL (LBSap and LBSApSal) with promising results in phase I and II trials (53–55). LBSap induced both humoral and cellular immune responses against *Leishmania infantum*, with high levels of total IgG and its subtypes (IgG1 and IgG2), expansion of circulating CD5+, CD4+, and CD8+ T lymphocytes as well as reduction of splenic parasite load (55).

One previous study designed to evaluate the quality of the Th1 response induced by *L. amazonensis* and *L. braziliensis* promastigotes extracts in PBMC from healed CL patients demonstrated that *L. amazonensis* response is associated with a low contribution of multifunctional T cells and a high number of IFN-γ single-positive effector cells, while *L. braziliensis* induces a Th1 response with high proportion of multifunctional T cells and low proportion of IFN-γ single-positive cells (39). As IFN-γ single-positive CD4+ T cells are short-lived, this can offer a possible explanation for the contrasting results observed in prophylaxis and immunotherapy studies with *L. amazonensis* whole-cell extract vaccine (Leishvacin^®^) (40, 56–58). The substantial amount of IFN-γ single-positive effector CD4+ T cells induced by this antigen may not be sufficient to induce long-term and good-quality protection against infection, but could be effective when a rapid and transient Th1 response is needed, as in the case of immunotherapeutic interventions. In addition, the capacity of *L. amazonensis* promastigotes extract to induce IL-10 secretion (59, 60), together with the generation of short-lived IFN-γ producing CD4+ T cells, could result in equilibrium between inflammatory and anti-inflammatory responses, allowing parasite killing and lesion resolution, as observed in the immunotherapeutic protocols tested so far.

If we combine the information that mice healed from a primary infection with *L. major* present the highest proportion of multifunctional CD4+ T cells and protection after a homologous challenge (38), together with the results obtained in healed CL patients after stimulation with *L. braziliensis* and *L. amazonensis* promastigotes extracts (39), we can consider the possibility that patients healed from *L. braziliensis* infection should display better protection to reinfection than *L. amazonensis* healed patients.

**Figure 1:**
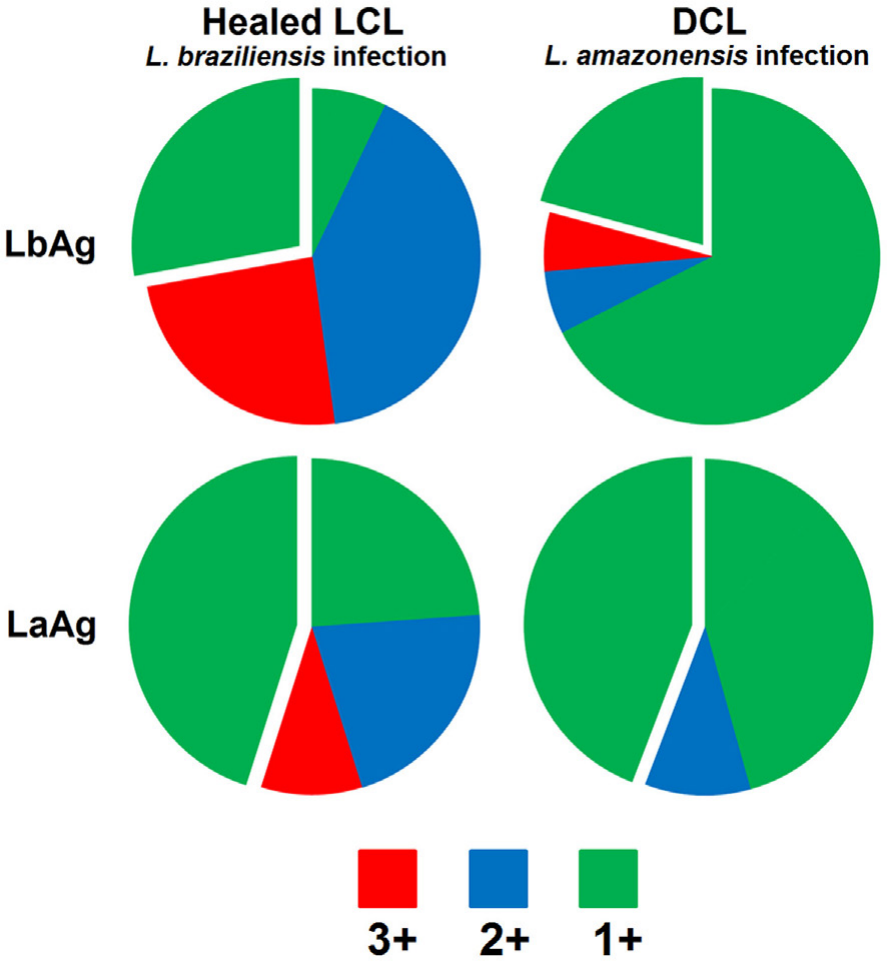
**Demonstrative figure of the CD4 T cell response induce in PBMC cultures of one patient healed from localized cutaneous Leishmaniasis caused by *L. braziliensis* (Healed LCL) in comparison to the response induced in PBMC cultures obtained from one patient with diffuse cutaneous Leishmaniasis caused by *L. amazonensis*, during remission of symptoms (DCL)**. Cells were stimulated “*in vitro*” with total promastigotes extracts form *L. braziliensis* (LbAg) or *L. amazonensis* (LaAg) and stained with monoclonal antibodies to determine the frequency of CD4 T cells expressing IFN-γ, IL-2, and TNF-α by multiparametric flow cytometry. Combination gates were applied to determine the percentage of cells that were able to produce any combination of these three cytokines. To determine the contribution of each phenotype to the total Th1 immune response analyzed the results are represented in the pie charts comprising cells expressing all three cytokines (in red – IFN-γ+TNF-α+IL-2+), any two cytokines (in blue – IFN-γ+TNF-α+IL-2−, IFN-γ+TNF-α−IL-2+, and IFN-γ−TNF-α+IL-2+), or any one cytokine (in green – IFN-γ+TNF-α−IL-2−, IFN-γ−TNF-α+IL-2−, and IFN-γ−TNF-α−IL-2+). Detached is the contribution of the IFN-γ+TNF-α−IL-2− single-positive cells phenotype. Data showed in this figure are part of a published study (39) approved by the National Ethical Clearance Committee of Brazil (CONEP), as well as by the Ethical Committee for Human Research from IPEC/FIOCRUZ, all of which adhere to the principles laid out in the Declaration of Helsinki. Informed consent was obtained from all participants.

It has never been reported that individuals that were infected with *L. braziliensis* or any other *Leishmania* specie are more susceptible to infection with *L. amazonensis*, but *L. amazonensis* infection does not give protection against a subsequent challenge with *L. braziliensis* or other *Leishmania* specie from the subgenus *Viannia*. On the other hand, recovery from *L. braziliensis* infection confers resistance to homologous challenge as well as to infection with *L. amazonensis* or *L. mexicana* parasites (6, 7). Interestingly, cells from DCL patients infected with *L. amazonensis* are able to differentiate into multifunctional T cells *in vitro* only after simulation with *L. braziliensis* promastigotes extract, while *L. amazonensis* stimulates high proportions of IFN-γ single-positive, terminal differentiated cells (Figure 1). This finding indicates that something intrinsic to *L. amazonensis* parasite antigens is responsible for the weak specific Th1 immune response observed during *L. amazonensis* infection (52, 61).

Even though parasites from the *Viannia* and *Leishmania* subgenera show highly conserved gene sequences with very few genes restricted to a given species (62–64), these similarities did not prevent different species from evolving some particularities related to the expression of virulence factors and the development of particular evasion mechanisms. Recently, the genome of *L. amazonensis* was sequenced and compared with other human pathogenic *Leishmania* spp. indicating that *L. amazonensis* and *L. mexicana* share groups of amastin surface proteins unique to the genus that could be related to specific disease outcomes. Additionally, a hypothetical interactome model of host protein and secreted *L*. (*L*.) *amazonensis* proteins revealed a possible interaction between an *L. (L.) amazonensis* heat-shock protein and mammalian Toll-like receptor 9 (65).

The low generation of multifunctional T cells induced by *L. amazonensis* can be one more factor, or, and most likely, can be a consequence of many others already described in the literature, implicated with the susceptibility to this *Leishmania* specie (23, 59, 65–73).

## Concluding Remarks

Prophylactic immunization is accepted as the most efficient and low-cost/benefit alternative to control infectious diseases. An ideal vaccine against Leishmaniasis must have several attributes: (1) safety, (2) accessibility for populations at risk, (3) induce long-lasting CD4 and CD8 specific T cell response, (4) be effective against *Leishmania* species responsible for visceral and tegumentary forms, (5) stability at room temperature to be used in the field, and (6) have prophylactic and therapeutic potential (74). Although it is possible to fulfill the attributes related to cost/benefit and safety, the development of a Leishmaniasis vaccine has proven a difficult goal to achieve. Not because of the discovery of candidate molecules, especially after the sequencing of the genome of different species of parasite, but rather because of the difficulties related to the still incomplete knowledge involving pathogenesis, the complex immune response needed for induction of protection, the lack of suitable experimental models, and the still fragmented knowledge about the development of immunological memory mechanisms.

Currently more than 30 *Leishmania* antigens have been or are being tested as candidate vaccines against visceral or tegumentary leishmaniasis. Many of them are very well conserved among different species of the parasite, but were not capable of inducing protection in clinical trials or are unable to protect against all species of the parasite. However, one study has demonstrated that heterologous protection is feasible, and associated with the presence of a “multifunctional Th1 response.” BALB/c mice immunized with a non-pathogenic *Leishmania donovani* parasite showed cross-protection against the challenge with *L. major* or *L. braziliensis*, and the immunization induced a long-term immune response characterized by high levels of multifunctional CD4 and CD8 T cells (48). Additionally, other authors observed a reduction in the frequency of parasitism in the bone marrow (54), as well as a reduction in splenic parasite loads (55) in dogs vaccinated against VL with LbSAP (a preparation of killed *L. braziliensis* promastigotes together with saponin), after *L. infantum* infection, although multifunctionality were not evaluated in those studies.

A point that also needs to be emphasized is that in natural infection, all the *Leishmania* species are co-deposited into the skin together with the vector saliva, and that saliva contains factors able to modulate the immune response (75–77). Studies have demonstrated that pre-exposure of sand fly saliva lead to either disease exacerbation (78, 79) or protection (80–83) upon *Leishmania* infectious challenge. Carregaro et al. (84) demonstrated that different inocula of *Lutzomyia longipalpis* salivary gland extract could modify the cellular immune response, reflecting in the pattern of susceptibility or resistance to *L. braziliensis* infection. It would be interesting to investigate whether a combination of saliva proteins with *Leishmania* proteins or extracts can shape the immune responses against infection, altering the quality of the immune responses by increasing the frequencies of multifunctional T cells. Moreover, the use of components that participate in the initial phase of infection could improve vaccine efficiency at the earlier stages of infection.

Certainly, there is still a long road ahead of us until an ideal Leishmaniasis vaccine be developed, but it is also undoubtable that multiparametric flow cytometry gave us a powerful tool to better evaluate correlates of protection and the development of memory T cell responses after infection and immunization. Since the crude and synthetic antigens tested so far were not able to induce consistent protection against *Leishmania* infections, it may be time to turn away our efforts from finding new candidate molecules, and focus on evaluating new presentation approaches of existing conserved molecules, specially the design of safe new adjuvants, that could direct the T cell-specific response toward long-lasting memory and multifunctional T cell phenotypes.

## Author Contributions

All authors listed have made substantial, direct, and intellectual contribution to the work and approved it for publication.

## Conflict of Interest Statement

The authors declare that the research was conducted in the absence of any commercial or financial relationships that could be construed as a potential conflict of interest.
